# Supplementary material for: Modelling the health labour market outlook in Kenya: Supply, needs and investment requirements for health workers, 2021–2035
Source: PLOS Glob Public Health. 2025 Jan 6;5(1):e0003966. doi: 10.1371/journal.pgph.0003966 (PMC11703116; doi:10.1371/journal.pgph.0003966)
Supplement: Attachment — Submitted filename: Response to peer review comments_4. 12.2024.docx [file pgph.0003966.s003.docx]

**Point-by-Point Response to Reviewer Comments/Feedback**

**The authors are extremely grateful to the esteemed reviewers** for their critical review, constructive suggestions and feedback which has greatly assisted us to improve the rigor and flow of the manuscript.

**Reviewer #1:**

**Comment**: All comments have been thoroughly addressed.

**Response**: We thank the reviewer for the constructive suggestions and feedback that helped us to improve the manuscript.

**Reviewer #2:**

**Comment:** My queries have been answered

**Response**: We are grateful to the reviewer for constructive suggestions and feedback that helped us to improve the manuscript.

**Reviewer #3:** 

**Comment 1**

In the discussion : the following is very cumbersome: suggest rewriting : As part of efforts to address some of these concerns and foster alignment between education and the needs of the health system, in 2021, Kenya held a conference to improve and harmonize health professions education curricula which led to actionable recommendations such as strengthening regulation and accreditation mechanisms, minimizing quick-fix trainings, and use of competency-based curricula.

Example: To address these concerns and enhance alignment between education and the needs of the health system, Kenya held a conference in 2021 focused on improving and harmonizing health professions education curricula. The conference resulted in actionable recommendations, including strengthening regulation and accreditation mechanisms, reducing reliance on short-term training programs, and implementing competency-based curricula.

**Response**: We are extremely grateful to the reviewer for the suggested edit. We have incorporated it in the revised manuscript.

**Comment 2**

Mistake noted:
As a result, it was not feasible to conduct sensitivity analysis which should be considered a limitation "if"should be replaced by "of " this paper.

**Response 2**: Thank you so much for spotting error. With gratitude, we have made the correction.
