# Supplementary material for: Modelling the health labour market outlook in Kenya: Supply, needs and investment requirements for health workers, 2021–2035
Source: PLOS Glob Public Health. 2025 Jan 6;5(1):e0003966. doi: 10.1371/journal.pgph.0003966 (PMC11703116; doi:10.1371/journal.pgph.0003966)
Supplement: Attachment — Submitted filename: Response to peer review comments_26.10.2024.docx [file pgph.0003966.s002.docx]

**Point-by-Point Response to Reviewer Comments/Feedback**

**Reviewer #1:** This was a comprehensive modelling study about Kenya’s healthcare workforce. This is an extremely important study for Kenya’s national healthcare planning. They used a published simulation tool and convened a technical expert panel to generate the necessary inputs. The equations underlying the models are well described.

I have just a few comments:
1) It would be helpful to know if the excel file used is a widely used model for HLMA and whether other models were considered.

**Response:** Thank you for the kind words and questions. The HLMA tool is widely used in Africa and beyond. It was developed by the authors following a rigorous process and to date has been applied in more than 15 countries across the world. The WHO HLMA support tool was published and launched this year as an instrument to operationalize the WHO HLMA guidebook. Available here: <https://iris.who.int/handle/10665/379059>

2) The supplementary excel file is very comprehensive, but it is a bit unclear where the inputs came from for every sheet. The addition of some footnotes would be helpful. In Table 1, are the data sources cites such at the reader can visit each of the respective websites/reports?

**Response:** Thank you for this feedback. Currently the tool is quite heavy, and we unfortunately had to sacrifice the detailed data source capturing so that it can be stable in excel. We are improving the tool, and the next version will capture it. We have thus, described the sources and triangulation methods in the methodology section (see Table 1).

3) “Despite progress, the WHO Africa Region needs more than 6.1 million alongside the maldistribution of available health workers, one of the most significant obstacles to achieving UHC by 2030” – consider rewording sentence to improve readability

**Response:** Many thanks for this feedback. This has been revised to read - Despite the progress in stock, the WHO Africa Region needs more than 6.1 million alongside addressing the maldistribution of the available health workers to achieve UHC by 2030

4) WHO Africa Region vs. WHO African Region – be consistent about Africa vs. African

**Response:** Thank you for this feedback. We have changed it to read WHO African Region across the document.

5) Introduction “absorption capacity” – define this in the introduction when it first used, as it may be unfamiliar to many readers

**Response:** Thanks for this feedback. We have included the definition in the introduction section.

6) Results, “range from 4.2% in 2021 to 2.7% by 2030” – Explain in the methods where this range comes from. Is this using a range of model inputs (like a sensitivity analysis using 80% to 125% the input values), or inputs from multiple sources? Is this by varying one input at a time, or all inputs simultaneously? Etc.

**Response:** Thank you for this comment. The range here refers to the variation in the projected growth rate over the years presented. We have removed the text referring to the range.

7) In general, were there sensitivity analyses that were performed within the Excel file model? Describe in methods.

**Response:** Thank you for this valid and important comment. Even though the tool used for the analysis is able to run sensitivity analysis (in the form of best and worst case scenarios) (Asamani et al., 2024), it relies on reported confidence intervals of the prevalence rates of diseases. However, available data at the time of analysis were point estimates without information on the boundaries of uncertainty. As a result, it was not feasible to conduct sensitivity analysis which should be considered a limitation if this paper. We have added this explanation as a limitation of the study. Future work will focus on mitigating this limitation.

**Reviewer #2:** Asamani et al. modeled the health labor market outlook in Kenya and found that there is a need for an increased health workforce and an increase in health workforce budget to employ these skilled workers.

Such analyses are important for countries to take stock and understand the projected needs and costs to permit early intervention, as these will only have future consequences.

I am not a modeler and cannot comment on the equations developed and used, although the methodology does seem intuitively plausible. The findings are relatively straight forward. For a general reader however there is some depth lacking that would be helpful to expand. The discussion is very short. There are therefore some clarifications that would be required:

1. Do the costs for the health care workers include the costs of training? If not, why not?

**Response:** Thank you for the positive feedback and for agreeing that this analysis is vital in health workforce planning at country level. The cost includes the cost for training health workers as shown in table 5 which estimates the economic feasibility of supply and needs compared with potential financial space. The costs for employing the projected supply, the need-based required health workers and training to meet the populations’ health needs are presented.

2. It is mentioned several times that a proportion of health care workers (nearly 1/3 mentioned in the introduction, 14% mentioned in the discussion – the numbers should be consistent throughout?) remain unemployed – please expand here, is this simply because of lack of funds to pay salaries or are there other drivers which may need to be considered in the models? Is this across all cadres or only some? If some, then which? This may be helpful to anticipate some redistribution of training resources

**Response:** Thank you for this important feedback. We have expanded the discussion section to extensively incorporate your very insightful suggestions about the causes of unemployment and how Kenya could tackle it. The 1/3 mentioned in the introduction is the overall level of health worker unemployment in the African region. However, 14% mentioned in the discussion is the estimated health worker unemployment in Kenya. We have clarified these in the revised manuscript. These estimates are based on a group of cadres and not particular cadre.

3. There is brief mention of concerns about the quality of nursing training – is this a funding issue or why is this? Are people being rushed through to meet a need? This would be counterintuitive if some nurses are remaining unemployed (unless unemployable)

**Response:** Thank you for the comment. Several factors have been highlighted including weak regulation and accreditation mechanisms, quick-fix trainings, and use of non-competency-based curricula. We have incorporated these in the revised manuscript.

4. Why does a country actively plan to export nurses if there is a need at home?

**Response:** Thanks for this comment. Migration of health workers, including nurses, is ongoing with some developed countries actively recruiting in the source countries. This impacts negatively on the health system of the source countries considering the investment in training. Thus, it is encouraged for countries to sign and implement bilateral agreements to regulate the migration of their health workforce for various reasons. Kindly refer to the Global Code on International recruitment of health workers.

5. In theory, if the health care workforce is well trained and adequate could one envisage the disease burden declining? How would this impact the modelling?

**Response:** Thanks for this comment. Yes, this will reduce morbidity and mortality and this is why the model takes into account the leading causes of disease burden, risk factors and interventions in the estimation.

6. Page 6, 2nd line of 1st paragraph, word missing “in the labour in the future”

**Response:** Thanks for this feedback. This has been corrected to read - how many health workers are anticipated to be in the health labour market in the future

7. Please suggest/hypothesise what an optimal approach/solution would be for Kenya to meet its own needs?

**Response:** Thank you for this comment. In the needs vs supply gap analysis subsection and table 4, we provided insights on the health workforce stock needed by Kenya if they are to adequately need the health needs of the population. Details on the financial implications are also presented in Table 5. Based on these, policy, plan and practice recommendations have been included in the discussion section. We have also provided extensive recommendations in the discussion section that would be useful for Kenya in addressing their gaps.

8. the Limitations are very focused, there are other potential sources of error in the data sets used, please mention these.

**Response:** Thanks for this feedback. We have expanded the limitations to include - Data was triangulated from various sources as highlighted in the methodology section. The limitations in these data sets are acknowledged and they also apply to this study. These limitations include variations in the data collection methods, rigor, frequency of updates, and levels of accuracy, which may lead to inconsistencies.

**Reviewer #3**: Observed grammatical/ typo mistakes

Page 4: - Over the last decade / then over the last decades – Perhaps be more concise for over the last decades – Suggest state – the years
**Response:** Thanks for this feedback. This phrase has been deleted.

page 5: 1. Grammar: "The descriptive aspects of the health labour market analysis has been discussed..." should be corrected to "have" .

page 13. Grammar
• "at annual average" should be "at an annual average."
• "the estimate 194,254" should be "the estimated 194,254."
• "are anticipated to by 26.5%" should be "are anticipated to increase by 26.5%."
page 16: Grammar
• “requirements for health workers in Kenya was projected at” → should be “were projected at.”
• "If the dynamics of the disease burden and population’s demographics remains..." → should be "populations' demographics remain..."

Page 17; Grammar/typos
• “The needs-based requirements for the nursing and midwifery workforce was projected...” → should be “were projected.”
• “A further 19% increase in the needed nurses and midwives id projected...” → “is projected.”
• “...projected from the the 2030 requirement...” → should be "the 2030 requirement

page 20
Grammar:
o “...if the disease burden burden is to be tackled” → Repetition of “burden.”
o “Without appropriate mechanisms to increase the throughput from the education pipeline, increase absorption and retention of the trained health workers, the projection show...” → should be "the projections show."
**Response:** Thanks for this feedback. These have been corrected.
Other:
Consider explaining/ defining the Need Availability Ratio (NAR) – as many readers will not be familiar with this
**Response:** Many thanks for this guidance. We have reflected the definition - NAR is a metric that compares the supply of the health workforce to the need, indicating the extent to which available health workers meet the healthcare needs of a population.

Recommendations - discussion
Discuss why there’s a high unemployment rate despite health workforce shortages and propose specific solutions to reduce it.

**Response:** Thanks for this guidance. This has been reflected in the discussion section.

I believe that the discussion should provide more insight and recommendation that would build on the information found in the models
For example:

1. More detail clarifying the roles of various stakeholders ( ex governments, private institution etc) in addressing the employment problems
2. Provide solutions or strategies for increasing training output and retention, which could be highlighted further, such as improving incentives for healthcare workers.
3. Discuss the roll that public/private partnerships may provide
4. Provide more detail on proposed strategies to close the 43$ funding gap and better integrate health workers into the work force - especially in rural and underserved areas
4. Page 4: The increase in the wage bill from KSh 4.97 billion to KSh 7.14 billion (2014–2019) More details are needed on why health outcomes have not met expectations despite these investments.- if known
5. Consider mentioning specific strategies to increase retention, such as government policies or funding for education, to provide actionable recommendations.
6 Highlight strategies for mitigating attrition and increasing training to meet health needs.

**Response:** Many thanks for these great insights and propositions. As advised, we have expanded the discussion section to address the points where evidence is available.

Potential risks: It would be nice to have insight in what the future concerns are:
1. Example: It may be helpful to highlight any potential risks or concerns if supply trends are not adjusted to meet future demands.

**Response:** Many thanks for this proposition. We have expanded the discussion section to address the points raised.
